# Lamb Wave-Based Damage Localization Feature Enhancement and Extraction Method for Stator Insulation of Large Generators Using VMD and Wavelet Transform

**DOI:** 10.3390/s20154205

**Published:** 2020-07-28

**Authors:** Ruihua Li, Jing Luo, Bo Hu

**Affiliations:** Department of Electrical Engineering, Tongji University, Shanghai 201804, China; 1832893@tongji.edu.cn (J.L.); bobo_hu@tongji.edu.cn (B.H.)

**Keywords:** damage location, lamb wave, stator insulation, variational mode decomposition, wavelet transform

## Abstract

Lamb waves are used to locate any damage in the stator insulation structure of large generators. However, it is difficult to extract the features of Lamb wave signals in a strong background noise environment, thus significantly reducing the accuracy with which the damage is located. This paper proposes a method based on variational mode decomposition (VMD) and wavelet transform to enhance and extract the location features of stator insulation damage signals of large motors. First, considering that the characteristics of VMD are sensitive to noise, the Lamb wave detection signal is decomposed, denoised, and reconstructed; the reconstructed signal is then wavelet-transformed to extract the time of flight (TOF) of the damage-scattered wave as the damage location feature; finally, the damage location is determined using the TOF features. The proposed method is experimentally tested and verified under various noise environments. The results show that the VMD and wavelet transform methods can significantly improve the signal-to-noise ratio of Lamb wave detection signals and the accuracy with which the damage is located under strong background noise. This study extends the applicability of Lamb wave-based non-destructive detection of stator insulation damage in complex environments.

## 1. Introduction

Stator winding is a core component of large generators. The insulation condition of the stator largely determines the life and operating reliability of such generators. The stator insulation structure gradually deteriorates because of the combined effect of electricity, heat, mechanical vibration, and environmental factors during its long-term operation, leading to various types of internal or surface damage and eventually causing insulation failure and fault shutdown [1,2,3,4]. Hence, timely and accurate detection of stator insulation damage can provide effective and reliable reference information for an early damage diagnosis [5].

Structural health monitoring (SHM) [6] has been employed for internal or surface damage detection in real time and early diagnosis and prediction, ultimately to avoid catastrophic accidents. A Lamb wave, which is a type of wave propagating in a plate-like structure, can be utilized to detect small damages in composite structures; Lamb waves have been applied to detect and locate stator insulation damage [7,8,9]. The multi-mode and dispersion characteristics of Lamb wave propagation, particularly in the laminated stator insulation composite structure of large generators, deteriorate the signal-to-noise ratio (SNR) and resolution of the sensing signal. In particular, in industrial environments, where noise interference is serious, the Lamb wave signal is weak, making damage feature extraction and signal analysis difficult. Therefore, the current focus is on the feature enhancement and extraction of Lamb wave non-destructive detection signals to more efficiently detect the damage in stator insulation structures.

In Lamb wave-based stator insulation damage detection, to reduce the impact of environmental noise on the Lamb wave signal, it is necessary to perform noise reduction processing on the received Lamb wave signal. Because of the nonstationary characteristics of Lamb waves, conventional noise reduction methods (such as linear filtering and nonlinear filtering) have some limitations in processing nonstationary signals. For nonstationary signals, wavelet threshold denoising has been applied [10]; however, the selection process of the wavelet basis function and threshold is complex, making it unsuitable for practical applications. By contrast, the empirical mode decomposition (EMD) method [11,12,13] does not require setting the basis functions in advance. It can perform adaptive decomposition based on the time-scale feature of the data itself. It can effectively handle nonlinear and nonstationary signals and suppress background noise. However, mode aliasing and over decomposition have been noted in practical applications. The ensemble empirical mode decomposition (EEMD) method [14] can to some extent compensate for the shortcomings of the EMD method; however, this method has the over decomposition problem. The VMD method [15] can be used to separate useful signals from noise signals by non-recursive variational decomposition and estimate the residual of the sub-component using a narrow-band Wiener filter. Hence, this method is suitable for the noise reduction of received signals in complex environments.

To locate the damage in a stator insulation structure, it is important to accurately extract the time of flight (TOF) features of the Lamb wave [9,16]. The multi-mode and dispersion characteristics of Lamb waves during propagation can hinder the extraction of TOF features. Li et al. [8] used the Hilbert transform to extract TOF features, albeit with a low location accuracy. A TOF extraction method based on the Hilbert cross-correlation coefficient was proposed in [9]. Although this method has a higher location accuracy than the Hilbert method, the TOF feature extraction ability under strong background noise is poor, thus limiting its practical applications. The wavelet transform method [17,18] has multi-resolution characteristics, can acquire the local features of a signal, and is suitable for the extraction of TOF location features. Therefore, this method has good application potential for TOF feature extraction from the stator insulation damage signals of large motors.

Based on the theory of non-destructive testing using Lamb waves, this paper proposes a method for enhancing and extracting the TOF features from stator insulation damage signals of large motors based on VMD and wavelet transform. The method can accurately identify the damage location. The rest of this paper is organized as follows. Section 2 presents the principle of identifying the damage location in a stator insulation structure based on Lamb waves. Section 3 introduces a noise reduction method applied to a Lamb wave damage signal based on the VMD and TOF location feature extraction method using wavelet transform. In Section 4, the effectiveness of the proposed method verified through experiments is evaluated. Section 5 gives a summary of the results.

## 2. Principle of Locating Insulation Damage Based on Lamb Waves

**Figure 1:**
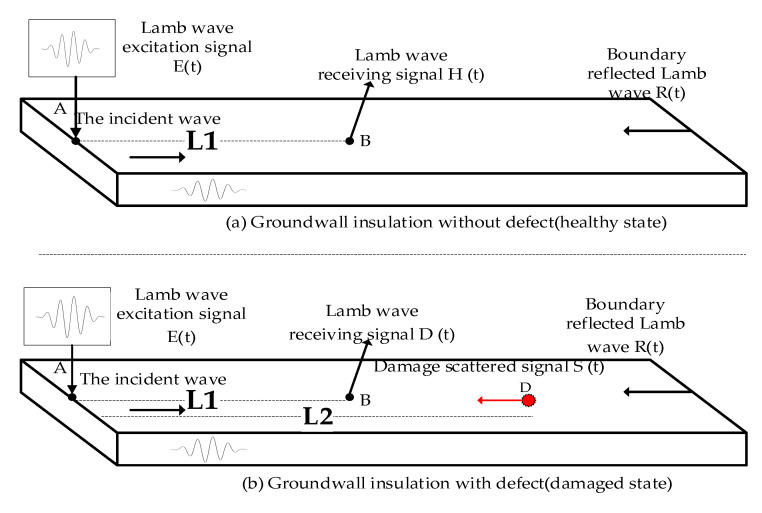
Schematic of stator insulation damage location based on Lamb wave.

In the health monitoring of insulation structures of large motor stators based on Lamb waves, accurately locating the stator insulation damage can provide important reference information for insulation state diagnosis and life assessment [19]. However, large generators typically operate in complex industrial environments with considerable noise interference, and the Lamb wave signal itself is relatively weak. Moreover, there are multi-mode and dispersion characteristics in the transmission process of the stator insulation structure, making it difficult to perform TOF feature extraction. Therefore, the extraction of the TOF features directly influences the accuracy with which the damage is located. Figure 1 shows the basic principle of using Lamb waves to locate stator insulation damage.

In Figure 1, piezoelectric ceramic (PZT) sensors are installed at points A and B for the excitation and reception of the Lamb waves, and the distance between points A and B is $L_{1}$. Point D is the damage location, and the distance from point A is $L_{2}$. Assuming that the stator insulation structure is in a healthy state, as shown in Figure 1, the received Lamb wave signal H(t) captured by the PZT at point B is mainly composed of the incident Lamb wave E(t) and boundary-reflected Lamb wave R(t), as illustrated in Equation (1):

(1) H(t)=E(t)+R(t)

As shown in Figure 1, if there is a damage at the stator insulation position *D*, the Lamb wave will scatter at the damage position based on its propagation characteristics. Under this condition, the received wave signal D(t) has three components, namely the incident Lamb wave E(t), boundary-reflected Lamb wave R(t), and damage-scattered Lamb wave S(t), which are defined as follows:

(2) D(t)=E(t)+R(t)+S(t)

According to Equations (1) and (2), the damage-scattered signal S(t) can be obtained as follows:

(3) S(t)=D(t)−H(t)

The damage location $L_{2}$ is determined by:

(4) $$TOF=t_{1}-t_{0}$$

(5) $$L_{2}=\frac{1}{2}(V_{g}\times TOF+L_{1})$$

where $t_{0}$ is the excitation time of the PZT at point A, $t_{1}$ is the arrival time of the damage-scattered wave at point B, and TOF is the TOF of the damage-scattered wave. The distance from the excitation point A to the reception point B is $L_{1}$, the distance from the damage point D to the point A is $L_{2}$, and $V_{g}$ is the average speed of the damage-scattered wave.

From Equations (4) and (5), we can infer that the stator insulation damage can be located as long as the TOF feature is accurately extracted when the distance from the excitation point to the receiving point and the speed of the scattered wave are known.

## 3. Lamb Wave Damage Signal Enhancement and Localization Feature Extraction Method

### 3.1. VMD-Based Noise Reduction Method for Damage Signal

The actual running state of the stator windings is complex and may be subject to severe noise interference. Noise reduction methods can improve the SNR of the received signal, and the TOF location features can thus be more accurately extracted. Due to the nonstationary characteristics of Lamb wave signals, conventional noise reduction methods have some limitations in processing Lamb wave signals. The VMD method can effectively deal with nonlinear and nonstationary signals and is therefore suitable for analyzing Lamb wave damage signals. This method can be used to adaptively analyze the damage signal and decompose it into different sub-components, so that the useful signals can be effectively separated from the background noise.

The main idea of the VMD method for blind source separation is to divide the original signal into different sub-components called band-limited intrinsic mode functions (BLIMFs), which can be expressed as:

(6) $$u_{k}(t)=A_{k}(t)\cos(\phi_{k}(t))$$

where $A_{k}(t)$ and $\phi_{k}(t)$ are the instantaneous amplitude and instantaneous phase of the BLIMF, respectively.

The BLIMF in Equation (6) is typically required to be a narrow-band signal. In this study, the Lamb wave signal is a narrow-band signal with a fixed center frequency. The actual received Lamb wave damage signal can be expressed as follows:

(7) D(t)=f(t)+p(t)+n(t)

where f(t) is the real damage signal of the Lamb wave, p(t) is the thermal noise interference of the large motor stator and rotor winding, i.e., white noise, and n(t) is the environmental noise due to electromagnetic interferences such as relay protection communication, radio communication, and carrier communication.

To extract the real damage signal f(t) from D(t), the separation between f(t) and noise is achieved as follows:

(8) $$\{{}_{s.t.\sum_{k=1}^{K}u_{k}(t)=D(t)}^{\underset{\left\{u_{k}\},\{\omega_{k}\right\}}{\min}\{{\sum_{k=1}^{K}\left\|\partial_{t}[(\delta (t)+\frac{j}{\pi t})*u_{k}(t)]e^{-j\omega_{k}t}\right\|}_{2}^{2}\},}$$

Equation (8) is a VMD decomposition expression. For one $u_{k}(t)$, which represents the real damage signal f(t) of the Lamb wave, $\left(\delta (t)+\frac{j}{\pi t})*u_{k}(t\right)$ is the unilateral frequency spectrum obtained by the Hilbert transform of $u_{k}(t)$. $\omega_{k}$ is the center frequency of $u_{k}(t)$, and the unilateral frequency spectrum can be modulated to the corresponding base band by mixing $e^{-j\omega_{k}t}$. The square normal form of the gradient of the modulated signal is calculated to obtain the bandwidth of the corresponding BLIMF sub-component. *K* is the number of BLIMF sub-components.

According to Equation (8), the received Lamb wave damage signal D(t) is composed of *K* BLIMF sub-components. *K* represents the number of different sub-components that the received Lamb wave damage signal is decomposed into. Generally, the larger the *K* is, the richer the damage signal types that the sub-components can represent. However, if *K* is too large, the decomposition of useful signal representing the real damage signal of Lamb wave will be over decomposed, which is not conducive to blind source separation. Generally, the more types of noise, the higher the value of *K*.

**Figure 2:**
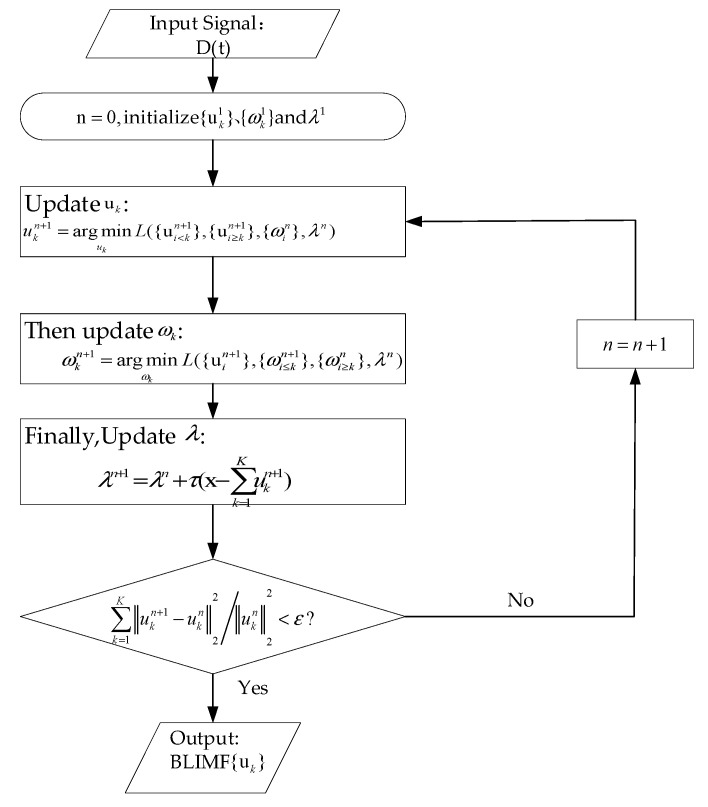
The alternating direction method of multipliers (ADMM) algorithm flowchart.

The following are the specific steps involved in the adaptive decomposition process through the VMD method:

i. The number *K* is set in advance, and by solving Equation (9), each BLIMF gets a limited bandwidth around its center frequency, and the total bandwidth is the lowest, so as to achieve the best separation effect.

(9) $$L(\left\{u_{k}\right\},\left\{\omega_{k}\right\},\lambda )=\alpha {\sum_{k=1}^{K}\left\|\partial_{t}[(\delta (t)+\frac{j}{\pi t})*u_{k}(t)]e^{-j\omega_{k}t}\right\|}_{2}^{2}+{\left\|x(t)-\sum_{k=1}^{K}u_{k}(t)\right\|}_{2}^{2}+\langle \lambda (t),x(t)-\sum_{k=1}^{K}u_{k}(t)\rangle$$

where α is the quadratic penalty term, and λ(t) is the Lagrangian multiplier. The term α ensures that the reconstructed signal maintains a high accuracy in the presence of noise. Generally, the larger α is, the narrower the bandwidth of the decomposed sub-components, and the less noise in the useful signal. However, if α is too large, it will cause the center frequency of the useful signal to not be correctly captured. In [20], α was set to 2000.

ii. The alternating direction method of multipliers (ADMM) algorithm can automatically find the saddle point of Equation (9); thus, the VMD method can adaptively decompose the received Lamb wave damage signal D(t). Figure 2 shows the flowchart of the ADMM algorithm. By constantly updating the values of $u_{k}$, $\omega_{k}$, and λ, ${{\sum_{k=1}^{K}\left\|u_{k}^{n+1}-u_{k}^{n}\right\|}_{2}^{2}/\left\|u_{k}^{n}\right\|}_{2}^{2}<\varepsilon$ is finally made to converge. Generally, ε is an arbitrarily small number greater than 0, normally set to 1e-6. For details, see [14].

Through the above algorithm, the real damage signal f(t) can be separated from the noise.

### 3.2. Wavelet Transform Extraction for TOF Location Features of Lamb Waves

As mentioned previously, the extraction of the TOF location features is key to locating the damage in the stator insulation structure. Because this structure is made of a composite material, and Lamb waves have multi-mode and dispersion characteristics, Lamb waves are prone to wave packet deformation and aliasing during propagation, which seriously affect the extraction accuracy of TOF location features.

Wavelet transform has multi-resolution characteristics. It can focus on the local features of the signal adaptively, which can help accurately extract the TOF features and improve the accuracy with which the damage is located. According to Equation (3), the damage-scattered signal S(t) can be extracted from the real damage signal f(t). The continuous wavelet transform of the damage-scattered signal S(t) with finite energy is defined as:

(10) $$W(a,b)=\frac{1}{\sqrt{a}}\int_{-\infty }^{+\infty }S(t)\Psi^{*}\left(\frac{t-b}{a}\right)dt$$

where W(a,b) is the wavelet transform coefficient, Ψ(t) is the mother wavelet function, $\Psi^{*}(t)$ is the complex conjugate function of Ψ(t), a is the scale variable, b is the time variable, and $\frac{1}{\sqrt{a}}\Psi \left(\frac{t-b}{a}\right)$ is the wavelet basis function generated by Ψ(t) through scaling and time translation.

The wavelet basis function in Equation (10) serves as an observation window in wavelet transform. It helps analyze the damage details such as the trend, discontinuity, and peak value of the lamb signal to be measured. Hence, the selection of the wavelet basis function is important. The complex Morlet wavelet has advantages such as the lack of endpoint effect and small amount of calculation [21]. In addition, the complex Morlet wavelet is a cosine signal that decays exponentially on both the left and right sides, and is similar to the freely attenuated Lamb wave signal waveform of the insulation structure; this method can achieve better matching. Therefore, the complex Morlet wavelet is selected as the wavelet basis function to perform continuous wavelet transform on the Lamb wave signal. The local features of the signal are thus amplified to accurately extract the TOF location features.

The energy spectrum is plotted after performing continuous wavelet transform on the Lamb wave signal using Equation (10), so that the damage characteristics of the Lamb signal can be analyzed more intuitively. The energy distribution equation for the Lamb wave damage-scattered signal in the time-scale domain is expressed as:

(11) $$E=\int_{b\geq 0}^{+\infty }\int_{a\geq 0}^{+\infty }|W(a,b){|}^{2}\cdot da\cdot db$$

With Equation (11), the energy spectra of the Lamb wave excitation signal E(t) and Lamb wave damage-scattered signal S(t) are plotted, to obtain the TOF location features and locate the damage in the large motor stator insulation structure.

**Figure 3:**
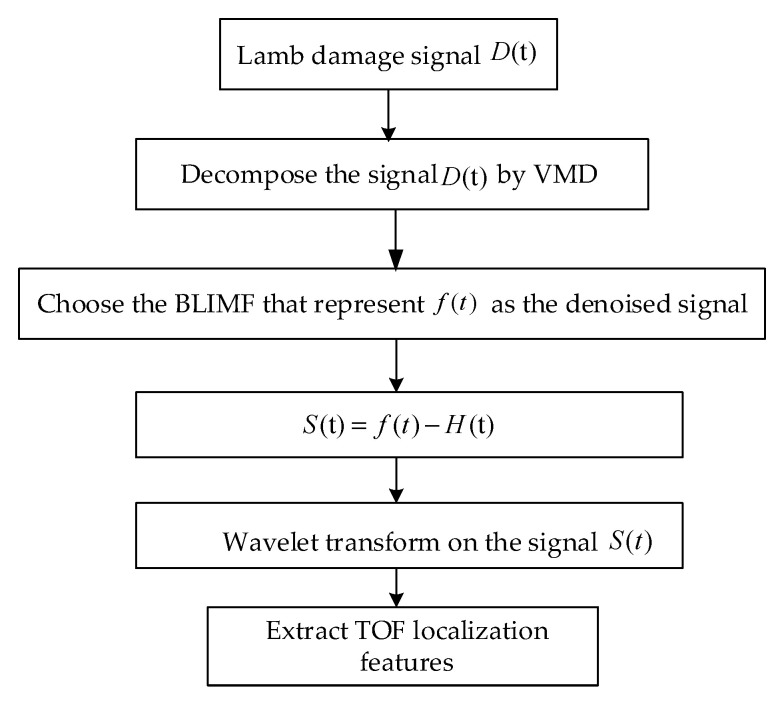
Flowchart of the time of flight (TOF) location feature enhancement and extraction algorithm.

Figure 3 shows the flowchart of the algorithm for enhancing and extracting the TOF location features of the large motor stator insulation structure based on VMD and wavelet transform.

## 4. Experimental Verification and Result Analysis

### 4.1. Experimental System

**Figure 4:**
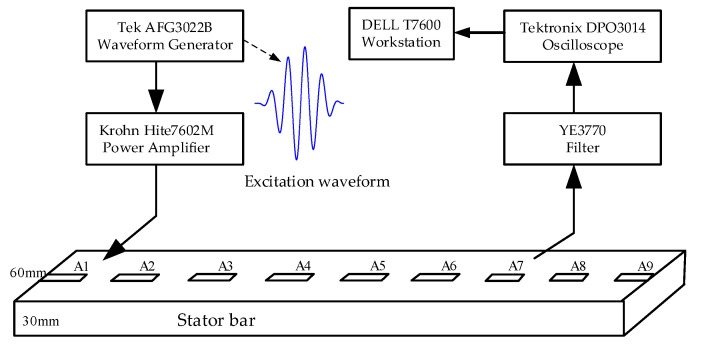
Structural diagram of the experimental system used for stator insulation damage detection.

To verify the feasibility and effectiveness of the feature enhancement and extraction method based on VMD and wavelet transform, an experimental system was set up, as shown in Figure 4.

In the experimental system, the sensor network composed of PZT ($A_{1}$-$A_{9}$) realizes the excitation and reception of the Lamb wave. The signal generator (AFG3022B) is used to generate the required Lamb wave excitation signal; the power amplifier (7602M) amplifies the Lamb wave excitation signal to drive the PZT to generate the Lamb wave; the filter (YE3770) filters the Lamb wave signal received by the PZT; the filtered signal is collected by the oscilloscope (DPO3014); Dell T7600 workstation is used to process and analyze the collected Lamb wave signal.

### 4.2. Experimental Results and Analysis

**Figure 5:**
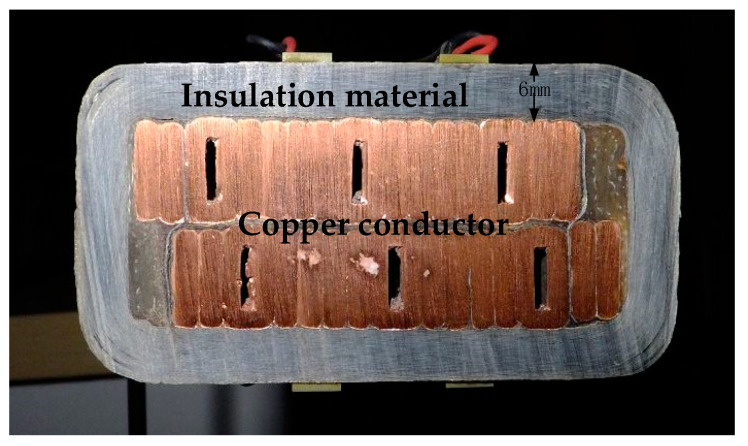
Sectional view of the stator bar.

The stator bar specimen used in this experiment is taken from an 18 kV/300 MW large motor. Figure 5 shows the cross section of the 60 mm × 30 mm stator bar with an insulation material thickness of 6 mm; which is molded and solidified by epoxy mica tape and wrapped around a copper conductor.

**Figure 6:**
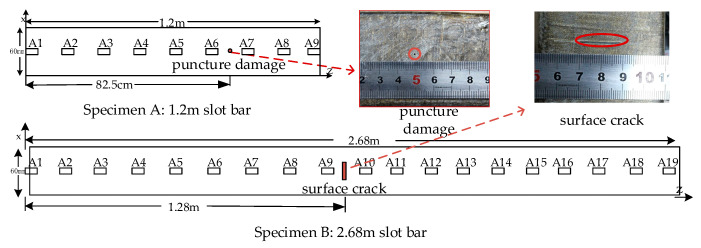
Experimental specimens.

The stator bar specimens used in the experiment are shown in Figure 6, including (a) stator bar specimen A: 1.2 m slot bar; (b) stator bar specimen B: 2.68 m slot bar.

As shown in Figure 6, a puncture damage with the diameter of 1 mm and the depth of 6 mm is induced approximately 82.5 cm from the left end of specimen A, and a surface crack damage with the length of 20 mm, the width of 2 mm, and the depth of 2 mm is induced approximately 1.28 m from the left end of specimen B. In [22], a five-cycle Hanning window sine pulse narrow-band signal with a center frequency of 13 kHz was used as the excitation signal to detect the stator insulation damage in the experiment.

#### 4.2.1. Test Results and Analysis for Puncture Damage

In the experiment, the puncture damage on the specimen A was tested first. To evaluate the effectiveness of the proposed algorithm, white noise and factory noise are introduced into the test and evaluation to simulate the real operating environment of the generator. The SNR is used to evaluate the intensity of the injected noise in the test:

(12) $$SNR=10\mathrm{lg}\frac{p_{s}}{p_{n}}$$

where $p_{s}$ is the average signal power, and $p_{n}$ is the average noise power.

**Figure 7:**
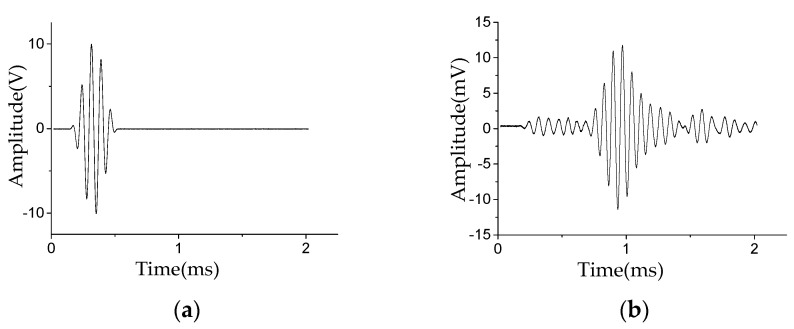
Signal waveforms: (**a**) Lamb wave excitation signal; (**b**) Lamb wave health signal *H*(*t*).

**Figure 8:**
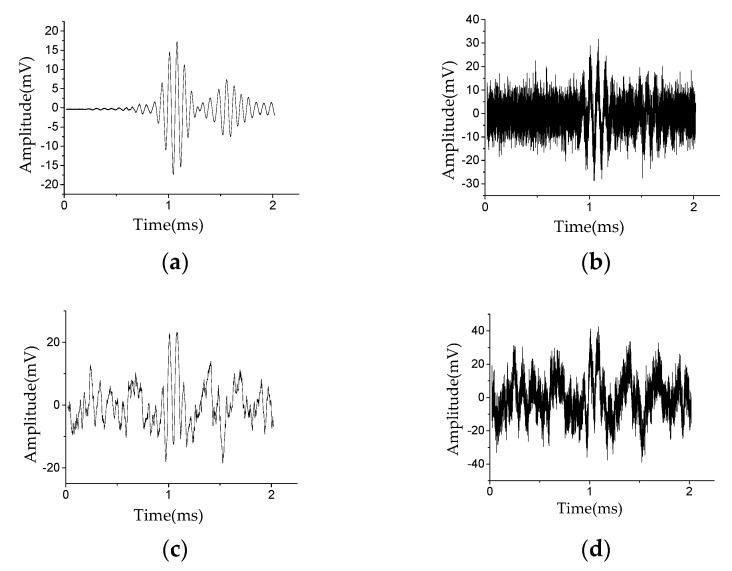
Waveforms of Lamb wave damage signal D(*t*): (**a**) pure damage signal; (**b**) damage signal with white noise; (**c**) damage signal with environment noise; (**d**) damage signal with mixed noise.

In this study, the effects of stator insulation damage location under different noise environments were tested. Figure 7a shows the Lamb wave excitation signal *E*(t). Figure 7b shows the Lamb wave health signal *H*(*t*), i.e., the Lamb wave signal received in the non-damaged state. Figure 8a shows the pure damage signal *D*(*t*) received in the damaged state. Figure 8b shows the damage signal after injecting white noise with an SNR of −3 dB. Figure 8c shows the damage signal after injecting environmental noise with an SNR of −3 dB. Figure 8d shows the damage signal after injecting mixed noise with an SNR of −3 dB, i.e., white noise with an SNR of −3 dB and environmental noise with an SNR of −3 dB. Compared with Figure 8a, the damage signal with white noise (Figure 8b) is almost submerged in the noise; the damage signal with environmental noise (Figure 8c) and the damage signal with mixed noise (Figure 8d) has been severely distorted. More information related to the stator insulation damage can be obtained by analyzing the Lamb wave damage signal.

**Figure 9:**
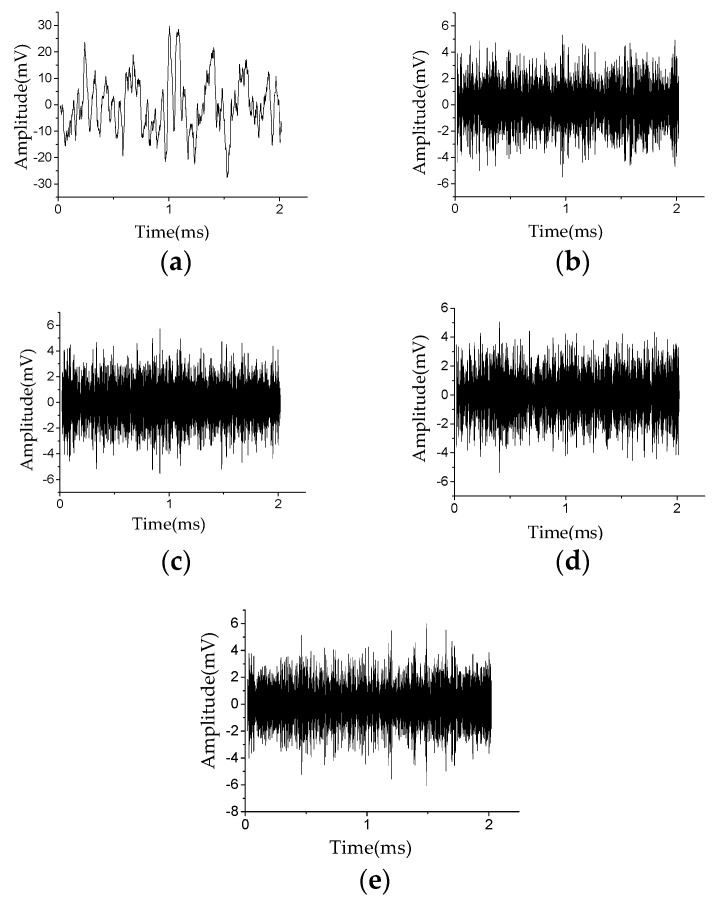
Waveforms of each band-limited intrinsic mode function (BLIMF) sub-component: (**a**) BLIMF1; (**b**) BLIMF2; (**c**) BLIMF3; (**d**) BLIMF4; (**e**) BLIMF5.

Second, the VMD method is used to further decompose the Lamb wave signal. As an example, the BLIMF sub-components are obtained after decomposing the Lamb wave damage signal with mixed noise (see Figure 8d), as shown in Figure 9. In practical application, the choose of K is usually related to the number of noise types. In this study, the impact of white noise and environmental noise on damage location is mainly considered. In order to avoid the over-decomposition of the damage signal (see Figure 8d), and to simulate the complexity of the generator operating environment as much as possible, the damage signal is decomposed into five sub-components.

Figure 9 shows that after VMD decomposition of the Lamb wave damage signal with mixed noise, the noise is separated into BLIMF2-5. According to the principle of VMD noise separation, the useful signal and noise are distinguished mainly based on the similarity between the sub-components and the original signal. This paper uses cross-correlation coefficients to determine the signal similarity between the sub-components and the original signal. The cross-correlation coefficient is the absolute value of the amplitude of the cross-correlation curve between the different subcomponents and the original signal. The greater the correlation coefficient, the higher the similarity. The cross-correlation formula [23] is expressed as:

(13) $$R_{fg}(\tau )=\int_{-\infty }^{+\infty }f(t)g(t-\tau )dt$$

where f(t) is the sub-component, g(t) is the original signal, and $R_{fg}(\tau )$ is the cross-correlation function of f(t) and g(t).

The correlation coefficient between different sub-components and the original signal calculated according to the cross-correlation function are listed in Table 1. It can be seen from Table 1 that BLIMF1 (Figure 9a) has the greatest similarity with the original signal (Figure 8d), so it is the real damage signal f(t) separated from the Lamb wave signal with mixed noise. Compared with Figure 8d, its peak value and waveform are clearer, which facilitates subsequent feature extraction.

**Figure 10:**
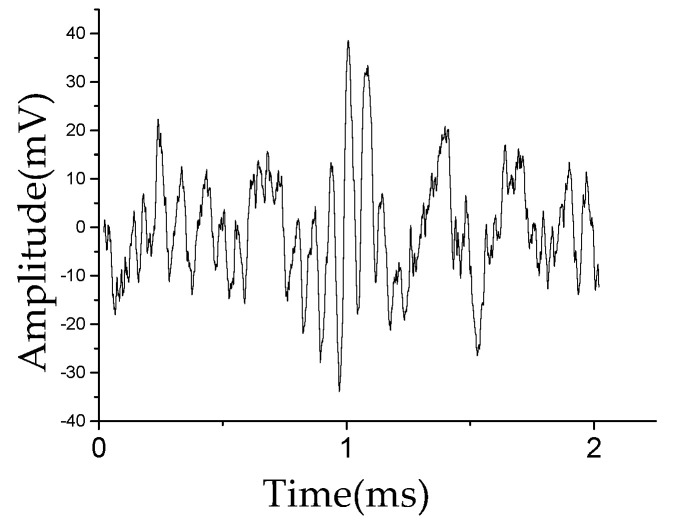
Lamb wave damage-scattered signal *S*(*t*) waveform.

**Figure 11:**
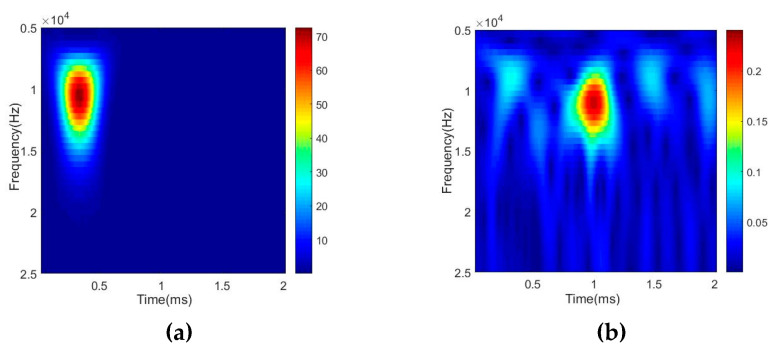
Energy spectra of (**a**) Lamb wave excitation signal *E*(*t*) and (**b**) damage-scattered signal *S*(*t*).

The wavelet transform is applied to further extract the damage location features. Figure 10 shows the Lamb wave damage-scattered signal obtained using Equation (3). Figure 11 shows the energy spectrum of the Lamb wave signal obtained using Equation (11), where Figure 11a,b show the energy spectra of the Lamb wave excitation signal E(t) and damage-scattered signal S(t), respectively.

Figure 11 shows that the peak arrival times of the Lamb wave excitation signal E(t) and damage-scattered signal S(t) are 0.3326 ms ($t_{0}$) and 1.0001 ms ($t_{1}$), respectively. From Equation (4), the TOF of the damage-scattered wave is determined to be 0.6684 ms. To effectively evaluate the effects of stator insulation damage location, Equation (14) is used to calculate and evaluate the accuracy with which the stator insulation damage is located.

(14) $$x_{re}=\left|\frac{x_{i}-x_{o}}{x_{o}}\right|\times 100\%$$

where $x_{re}$ is the relative error, $x_{i}$ is the distance between the damage calculation position and the excitation end, and $x_{o}$ is the distance between the actual damage position and the excitation end.

According to Equation (5), the identified location $L_{2}$ of the stator insulation damage is 88.06 cm, the actual damage location is 82.5 cm, the absolute error is 5.56 cm, and the relative error is 6.74%.

To effectively evaluate the noise reduction effect of the VMD method based on the wavelet transform feature extraction method, the effects of stator insulation damage location before and after noise reduction are compared under different noise environments. The statistics of the damage location results under different noise environments are listed in Table 2.

As listed in Table 2, the relative error after noise reduction using the VMD method is significantly improved. Thus, the applicability of Lamb wave-based non-destructive testing can be extended to complex noise environments.

As mentioned previously, the extraction of the TOF features directly influences the accuracy with which the damage is located. In addition to noise, which affects the accuracy of TOF extraction, the damage feature extraction methods influence the accuracy of TOF extraction. To effectively evaluate the effect of TOF feature extraction and damage location based on wavelet transform, the damage location effect of the proposed method is compared with that of the Hilbert-based [8] TOF feature extraction method after noise reduction of the Lamb wave signal in different noise environments. Because the peak moment of the signal envelope represents the moment when the signal energy is most concentrated, the Hilbert-based method can obtain the TOF feature by making difference between the envelope peak moment of the extracted damage-scattered signal and the excitation signal. The statistics of the damage location results of the different TOF feature extraction methods are listed in Table 3.

As listed in Table 3, compared with the Hilbert method, the wavelet transform method can better extract the TOF location features, thereby effectively improving the location accuracy. Hilbert-based method only obtains TOF by extracting signal envelope. Although it is simple and convenient, its biggest disadvantage is poor anti-interference and stability. The wavelet transform feature extraction method uses the wavelet basis function as the observation window, which can more accurately observe the changes of the waveform and extract detailed information such as signal change trends and peak values, so the extracted TOF features are more accurate. At the same time, the wavelet transform method itself also has anti-interference ability, and can cooperate with the VMD method to form a better anti-noise effect, so the TOF feature extracted by the wavelet transform method is more accurate and the location accuracy will be higher.

#### 4.2.2. Surface Crack Damage Location Test Results and Analysis

Surface cracks are another type of typical damage in stator insulation structures. If they can be detected, early diagnosis and prediction can be carried out, and timely response measures can be taken, which has high practical value. As with specimen A, a comparison test was performed on the surface crack damage location in specimen B under different noise environments. The results of the damage location test are listed in Table 4 and Table 5, respectively.

From Table 4 and Table 5 similar conclusions can be made. The VMD method combined with wavelet transform can effectively improve the insulation damage location accuracy of large motor stators, making this approach quite suitable for the detection of stator insulation damage.

## 5. Conclusions

The actual working environment of large motor stator windings is relatively complicated, and it is susceptible to severe noise interference. Moreover, Lamb waves have multi-mode and dispersion characteristics when propagating in the stator insulation composite material, making it difficult to locate the damage. Improving the SNR of the received signal and accurately extracting the TOF location features are key to performing accurate detection. This paper proposes a method for enhancing and extracting the damage localization features based on VMD and wavelet transform. Verification experiments are performed with two types of typical damages: puncture damage and surface crack damage.

The experimental results show that the VMD method can effectively improve the SNR of the received signal and overcome the problems due to the relatively weak useful signal and difficulty in extracting it under strong background noise, thereby extended the application range of the detection method employed for stator insulation damage. The wavelet transform method can effectively overcome the influence of the Lamb wave dispersion effect, accurately extract the TOF location features, and further improve the location accuracy of stator insulation damage. The proposed method based on VMD and wavelet transform can detect the damage that occurs during the stator insulation aging process and locate the damage location. It can be used as an effective condition monitoring tool for large generators in actual operating environment to supplement existing methods. The proposed approach significantly extends the applicability of non-destructive detection using Lamb waves. In future work, we will research how to combine the proposed method with non-invasive detection methods, which is more conducive to practical industrial applications.

## Figures and Tables

**Table 1:** Statistics of the correlation coefficient between different sub-components and the original signal.

Sub-Component Category	BLIMF1	BLIMF2	BLIMF3	BLIMF4	BLIMF5
**cross-correlation coefficient**	0.88	0.21	0.21	0.21	0.21

**Table 2:** Statistics of damage location results of specimen A before and after noise reduction using VMD in different noise environments.

Noise Type	Damage Location (cm)	Specimen A			
		Without Denoise		VMD	
		Identified Location (cm)	Relative Error (%)	Identified Location (cm)	Relative Error (%)
10 dB white noise	82.50	90.25	9.39	86	4.24
5 dB white noise	82.50	90.64	9.87	86.29	4.59
−3 dB white noise	82.50	91.01	10.32	87.82	6.45
10 dB environment noise	82.50	91.09	10.41	86.08	4.34
5 dB environment noise	82.50	92	11.52	86.14	4.41
−3 dB environment noise	82.50	95.1	15.27	87.75	6.36
10 dB mixed noise	82.50	91.24	10.59	86.78	5.19
5 dB mixed noise	82.50	92.15	11.70	87.37	5.90
−3 dB mixed noise	82.50	98.2	19.03	88.06	6.74

**Table 3:** Statistics of damage location results of specimen A under different feature extraction methods.

Noise type	Damage Location (cm)	Specimen A			
		Hilbert		Wavelet Transform	
		Identified Location (cm)	Relative Error (%)	Identified Location (cm)	Relative Error (%)
10 dB white noise	82.50	90.68	9.91	85.66	3.83
5 dB white noise	82.50	90.98	10.29	86.21	4.50
−3 dB white noise	82.50	91.21	10.56	86.84	5.26
10 dB environment noise	82.50	86.51	4.86	85.92	3.42
5 dB environment noise	82.50	86.66	5.04	86.15	4.42
−3 dB environment noise	82.50	88.26	6.98	87.43	5.98
10 dB mixed noise	82.50	90.83	10.09	86.54	4.90
5 dB mixed noise	82.50	91.29	10.65	87.47	6.02
−3 dB mixed noise	82.50	91.74	11.2	88.2	6.91

**Table 4:** Statistics of damage location results of specimen B before and after noise reduction using VMD in different noise environments.

Noise Type	Damage Location (cm)	Specimen B			
		Without Denoise		VMD	
		Identified Location (cm)	Relative Error (%)	Identified Location (cm)	Relative Error (%)
10 dB white noise	140.00	133.30	4.79	134.88	3.66
5 dB white noise	140.00	132.11	5.64	134.78	3.73
−3 dB white noise	140.00	131.97	5.74	134.45	3.97
10 dB environment noise	140.00	133.50	4.64	135.07	3.52
5 dB environment noise	140.00	133.08	4.94	134.79	3.72
−3 dB environment noise	140.00	132.87	5.09	133.75	4.46
10 dB mixed noise	140.00	133.08	4.94	134.66	3.82
5 dB mixed noise	140.00	132.53	5.34	134.31	4.07
−3 dB mixed noise	140.00	131.14	6.33	133.13	4.91

**Table 5:** Statistics of damage location results of specimen B under different feature extraction methods.

Noise Type	Damage Location (cm)	Specimen B			
		Hilbert		Wavelet Transform	
		Identified Location (cm)	Relative Error (%)	Identified Location (cm)	Relative Error (%)
10 dB white noise	140.00	133.49	4.65	134.88	3.66
5 dB white noise	140.00	133.03	4.98	134.78	3.73
−3 dB white noise	140.00	131.82	5.84	134.45	3.97
10 dB environment noise	140.00	133.83	4.41	135.07	3.52
5 dB environment noise	140.00	133.62	4.56	134.79	3.72
−3 dB environment noise	140.00	147.53	5.38	133.75	4.46
10 dB mixed noise	140.00	133.48	4.66	134.66	3.82
5 dB mixed noise	140.00	147.14	5.10	134.31	4.07
−3 dB mixed noise	140.00	148.44	6.03	133.13	4.91
